# A Diabetes Education App for People Living With Type 2 Diabetes: Co-Design Study

**DOI:** 10.2196/45490

**Published:** 2023-09-18

**Authors:** Anita Pienkowska, Chin-Siang Ang, Maleyka Mammadova, Muhammad Daniel Azlan Mahadzir, Josip Car

**Affiliations:** 1 Center for Population Health Sciences Lee Kong Chian School of Medicine Nanyang Technological University Singapore Singapore

**Keywords:** diabetes, health apps, co-design, chatbot, type 2 diabetes, self-management, mobile health, mHealth, chronic disease, digital education, mobile phone

## Abstract

**Background:**

Type 2 diabetes (T2D) is a growing global health concern, including in Singapore. Diabetes education programs have been shown to be effective in improving health outcomes and diabetes self-management skills. Mobile health apps have emerged as useful tools for diabetes education; however, their use and acceptance by the target population remain inconsistent. Therefore, end-user participation in the design and development of a mobile health app is crucial for designing an acceptable app that can improve outcomes for populations with a chronic disease.

**Objective:**

The objective of this study was to apply an end-user participatory approach to co-design a diabetes education app prototype for people living with T2D by exploring their perceptions, acceptance, and usability of an app prototype, as well as their diabetes experience and perspectives on digital diabetes education.

**Methods:**

A total of 8 people with T2D, who were recruited from diabetes management Facebook groups, participated in 4 web-based surveys via Qualtrics and 2 structured interviews via Zoom (Zoom Video Communications, Inc) between August 20, 2021, and January 28, 2022. Descriptive statistics and thematic analyses of the discussion and iterative feedback on the app prototype were used to assess the participants’ perceptions of living with T2D, attitudes toward digital diabetes education, and acceptance of the prototype.

**Results:**

Analyses of the surveys and interview data revealed 3 themes: challenges of living with T2D; validation, acceptability, and usability of the diabetes education app prototype; and perspectives on digital diabetes education. In the first theme, participants highlighted the importance of solitary accountability, translating knowledge into practice, and developing pragmatic self-consciousness. The second theme indicated that the diabetes education app prototype was acceptable, with information and appearance being key; revealed ambivalent and polarized opinions toward the chatbot; and confirmed potential impact of the app on diabetes self-management skills and practice. The third theme comprised the necessity of using a variety of information-seeking strategies and recommendations for desired content and app qualities, including accessibility, adaptability, autonomy, evidence-based design and content, gamification, guidance, integration, personalization, and up-to-date content. The findings were used to reiterate the app design.

**Conclusions:**

Despite a small sample size, the study demonstrated the feasibility of engaging and empowering people living with T2D to consider digital therapeutics for diabetes self-management skills and practice. Participants gave rather positive feedback on the design and content of the app prototype, with some recommendations for improvements. The findings suggest that incorporating end-user feedback into app design can lead to the creation of feasible and acceptable tools for diabetes education, potentially improving outcomes for populations with a chronic disease. Further research is needed to test the impact of the refined diabetes education app prototype on diabetes self-management skills and practice and quality of life.

## Introduction

### Background

The increasing prevalence of type 2 diabetes (T2D) is a global concern, including in Singapore, where 1 in 10 adults has the condition [1]. The projections suggest that, by 2050, diabetes will affect 1 in 6 Singaporean adults [2]. Individuals require lifelong education and support to feel empowered to successfully manage their diabetes condition [3-6]. Diabetes education has been demonstrated to improve health outcomes and quality of life, as well as increase appropriate health care use [7-9]. The more time spent on diabetes education, the better the health outcomes [9]. However, current programs are resource intensive and costly [10], resulting in a lack of immediacy, timeliness, and flexibility of access. Two out of 5 people living with diabetes report that they have not received diabetes education [11].

To address this gap, scalable digital health approaches, such as mobile health apps, are increasingly gaining attention and relevance [12,13]. With 100% mobile phone ownership in Singapore [14], patient-oriented technology can be a viable option for complementing traditional health services and actively involving people living with T2D in health decision-making related to sustainable lifestyle changes [15,16]. There is a growing body of evidence linking patient participation in product design and development to efficacy and clinical safety [17-19], prompting a shift from passive participation to collaboration, known as co-design, cocreation, or coproduction [20-22]. This approach aims at ensuring that the end product caters to the target audience by recognizing their unique experience and needs.

Co-design can be conducted in the predesign phase, generative phase, or evaluative phase to produce a product or service that is then tested in the postdesign phase [23,24]. The predesign phase aims at understanding people’s needs, the generative phase at producing ideas and concepts, the evaluative phase at assessing the effects of the product or service, and the postdesign phase at investigating how people experience the product. In comparison with a user-centered yet noncollaborative tool such as user personas (a fact-based visual representation of average primary users that describes demographics, pain points, and challenges) [25], co-design allows for a much more active contribution to product design and development.

### Objectives

In this study, we aimed to assess the initial acceptability and usability of a diabetes education app prototype using a participatory experience-based approach. Our goal was to use co-design findings to inform the development of the app planned for further evaluation in the postdesign phase. Specifically, the objectives of the study were as follows:

- Aim 1 (A1): understand the challenges of diabetes management from the perspective of people living with the condition
- Aim 2 (A2): validate the design of a diabetes education app prototype
- Aim 3 (A3): understand perspectives on digital diabetes education

## Methods

### Study Design

**Figure 1:**
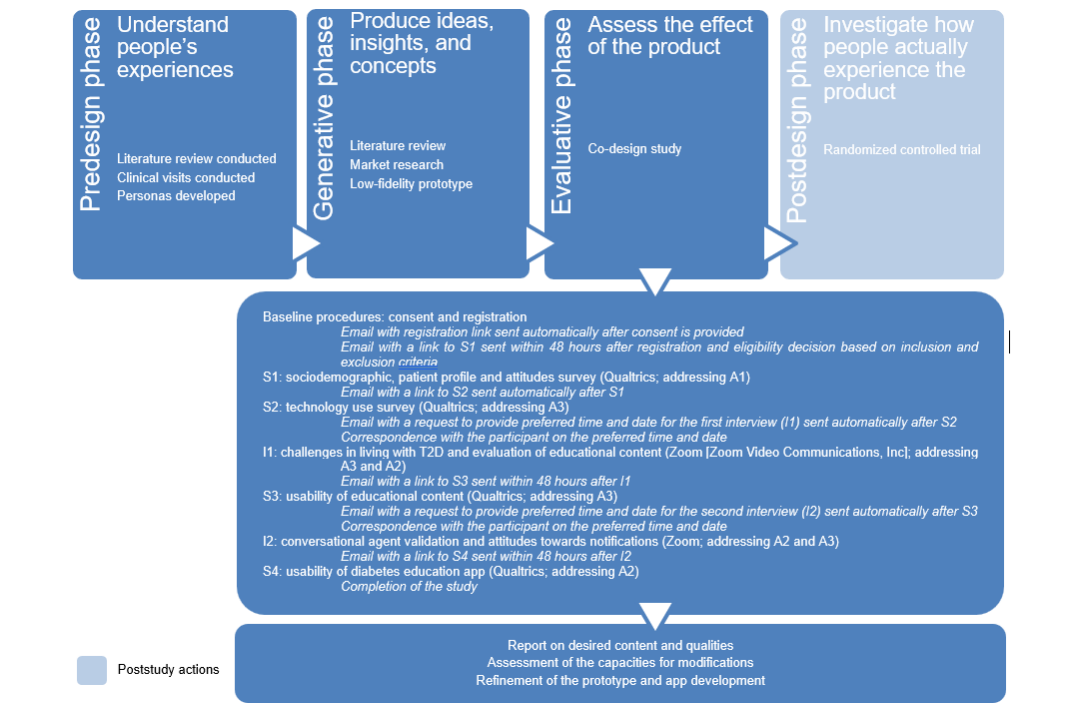
Flow of data collection. A1: aim 1; A2: aim 2; A3: aim 3; S1: survey 1; S2: survey 2; S3: survey 3; S4: survey 4.

Using participatory methods, this study reports on the co-design of an educational app to support people living with T2D by including user input at an early stage of app design and development. We co-designed the app prototype (Multimedia Appendix 1) in the evaluative phase of app design to inform further app development [23,24] (Figure 1). The purpose of this co-design was to explore (A1) diabetes management challenges; (A2) the app prototype, its content, and features; and (A3) digitalized diabetes education through 4 surveys and 2 interviews to produce insights for subsequent app prototype iterations and development. The app will be further tested in the postdesign phase. The sequence of the instruments was designed to gather information on participants’ characteristics and profile (survey 1 [S1] and survey 2 [S2]) during the initial contact. Next, to understand participants’ experiences of living with T2D and to evaluate the educational content, we used open-ended questions (interview 1 [I1]), and to assess usability, we used closed-ended questions (survey 3 [S3]). Subsequently, we evaluated participants’ perceptions on a conversational agent and notifications prototype through open-ended questions (interview 2 [I2]) and assessed usability through closed-ended questions (survey 4 [S4]; Figure 1).

This study followed the COREQ (Consolidated Criteria for Reporting Qualitative Research) guidelines [26] (Multimedia Appendix 2).

### Ethics Approval

This study received ethics approval from the Nanyang Technological University Institutional Review Board (IRB-2020-04-010).

### Study Participants and Recruitment

Eligibility for the study included Singaporean citizens, permanent residents, and foreign domestic workers aged ≥21 years with T2D who were English speakers; owned a PC, tablet device, or smartphone; and could use the app prototype. Those diagnosed with type 1 diabetes, those caring for people living with type 1 diabetes, and individuals unable to provide consent were excluded.

Participants were recruited from diabetes-related social media pages and groups (ie, Facebook). Individuals interested in participating in the study clicked on a link directing them to a study information sheet and consent form in Qualtrics. Each participant signed a digital informed consent form that included details about the study procedures, confidentiality, and data management. The consent form also included specific statements that provided approval to collect audio and video recordings of the interviews. After providing consent, each individual received an automatic message with a link to the registration form, which comprised questions regarding the inclusion and exclusion criteria. The responses were evaluated by the team within 48 hours, and if the inclusion criteria were met, the participant received an email with a link to the first survey (S1). All participants met the inclusion criteria for the study.

In total, 11 participants signed the consent forms: 8 (73%) began and completed the study, whereas 3 (27%) did not respond to 2 emails informing them about the next steps. It is unclear whether there is a difference between those who dropped out and those who completed the study, but the eligibility criteria aimed to create a homogeneous sample. Differences, if any, may be due to other factors. We expected to recruit at least 8 participants in the study, based on a previous co-design study that used a similar approach [27]. As subthemes were repeated in the data obtained, data saturation was determined to have been achieved.

### Diabetes Education App Prototype

The diabetes education app prototype was designed to include a sample of educational materials and to showcase features designed to facilitate learning and behavior change. The education module comprised 4 units, each consisting of conversation prompts, learning cards, self-reflection cards, quizzes, and a microchallenge. In addition, the education module was accompanied by chatbot conversations, including preunit conversation prompts as well as frequently asked questions and postmodule action-oriented conversations. The low-fidelity flat prototype of the educational modules was designed in Adobe XD, whereas the chatbot conversations were developed partially in Adobe XD and partially in Quriobot for active participation in testing. However, the nature of the low-fidelity flat prototype did not allow all features to be connected in one homogeneous flow. Thus, during the interview, the interviewer explained the context for each tested feature. Examples of the materials are presented in Multimedia Appendix 1.

The presented materials centered on foot care for people living with T2D and were designed based on clinical guidelines such as those from Singapore’s ministry of health [4], the Singapore Agency for Care Effectiveness [28], and the International Working Group on Diabetic Foot [29]. Additional sources included academic papers [30], practical guides from medical centers (the University of Vermont Medical Center [31] and Mayo Clinic [32]), and health promotion leaflets from the Tan Tock Seng Hospital in Singapore. The final content was reviewed for medical accuracy by in-house doctors, diabetes educators, and podiatrists from the Tan Tock Seng Hospital.

The content development process used an instructional design approach to develop educational materials in a consistent, dependable, and learner-centered manner [33]. The process included creating personas with learning needs, priorities, attitudes, motivations, and pain points [25], as well as learning objectives for competency development, including knowledge, skills, and attitudes [34,35]. To increase perceived meaningfulness and relevance [36], the content was translated into practice-oriented learning cards, quizzes, and conversations, using findings from web forums such as Diabetes UK Support Forum, and written to address the persona’s pain points. Furthermore, the information design followed microlearning rules to make the educational material repetitive, reflective, and constructivist [37]. The content adhered to the multiple representation principle and cognitive load theory, highlighting the importance of using both words and pictures rather than just words [38,39], that is, a picture has been created to illustrate the message conveyed every 2 to 3 sentences. The copywriting style followed empathetic language principles [40-42] and the Patient Education Materials Assessment Tool [43].

To encourage users to take action, motivational interviewing [44,45] and nudging [46] constructs were incorporated into the learning cards, quizzes, and conversations. The components of the health belief model (perceived severity, susceptibility, benefits, barriers, and self-efficacy) [47,48] informed the elements of various content and features, such as learning cards, quizzes, and action-oriented conversations, to raise awareness about possible flawed beliefs and assumptions [47,48]. The connections between the app’s features and the sequence of user actions were developed and mapped to initiate behavior change and followed the transtheoretical model [49] and behavior change techniques [50].

### Data Collection

Data were collected through 4 web-based surveys via Qualtrics (S1, S2, S3, and S4) and 2 remote interviews via Zoom (Zoom Video Communications, Inc; I1 and I2). Figure 1 depicts the flow of data collection. The study commenced with 2 baseline surveys: the first survey (S1) aimed to collect participants’ sociodemographic characteristics, health profile (eg, age at diagnosis, current treatment, complications, and frequency of physician consultations), experiences with diabetes education programs, and health beliefs. The second survey (S2) comprised questions regarding participants’ use of technology as well as their acceptance of, and attitudes toward, diabetes management apps. Subsequently, the first interview (I1) was conducted to investigate participants’ perceptions of the low-fidelity flat prototype and their attitudes toward behavior change in the context of diabetes self-management. After I1, a survey (S3) was administered to evaluate the usability of the educational content. The survey included questions adapted from the health information technology acceptance model (HITAM) [51] and the Mobile Application Rating Scale (MARS) [52] to examine the perceived usefulness and ease of use of the prototype. The second interview (I2) focused on the presentation of an interactive chatbot conversation in Quriobot and participants’ attitudes toward nudges in the form of notifications. This was followed by a survey (S4) that included questions adapted from the HITAM [51] and the MARS [52] to analyze the perceived usefulness and ease of use of the rules-based interactive conversational agent.

The interviews were conducted between August 20, 2021, and January 28, 2022 at participants’ preferred times and availability. There was an interval of 3 to 14 days between the first and second interviews. The interviews were audio and video recorded using a built-in recorder on the Zoom platform. Each participant completed 2 interviews that lasted between 30 minutes and 1 hour. The interviews were conducted by AP (14 interviews), a female research fellow, and MM (2 interviews), a female research assistant; both were provided with training on qualitative research and on conducting interviews, including mentorship provided by JC, and both went through mock interviews before the study commencement. Neither interviewer had any prior knowledge of the participants before the study, and nor did the participants have any knowledge about the interviewers. An interview guide was developed to assist the interviewers in acquiring information related to the attitudes toward challenges in diabetes management and perceptions of diabetes education app prototype functionality and design (refer to the interview questions in Multimedia Appendix 3). Interviewers took field notes to highlight essential topics and nonverbal cues.

### Data Analysis

Initial descriptive statistics were calculated to describe the data from 2 baseline surveys and summarize the characteristics of the study participants. Frequency analyses were used to examine closed-ended questions from S3 and S4. The findings from S3 and S4 have been summarized in the subsection Validation, Acceptability, and Usability of the Prototype and, where possible, compared with the findings from thematic analysis.

Reflexive thematic analysis was used to uncover key themes from the data collected from the interviews [53]. To ensure accuracy, the automated transcripts generated by Zoom were compared with the original recordings and corrected accordingly. The transcripts were then deidentified. The deconstruction and analysis of the interview transcripts were carried out using Microsoft Excel by AP, who thematically coded the transcript using data-driven codes. The codes were then organized into higher-order themes. A code dictionary was created using a hybrid deductive and inductive approach, whereby codes were defined based on the research questions (deductive coding) as well as through familiarization with the data; generating initial codes; searching for patterns; grouping the codes into inductive themes; and reviewing, regrouping, and renaming subthemes (inductive coding; Multimedia Appendix 4). The themes were refined through discussion between AP and CSA. The analyses from surveys and interviews were then integrated to uncover the participants’ perceived challenges of living with T2D, their acceptance of the app prototype, perspectives on digital diabetes education, and the desired features and qualities of the app. These desired features and qualities have been collated and evaluated against budgetary and time constraints. The findings, which had an impact on the subsequent app prototype design and app development carried out by a vendor, have been summarized in the App Prototype Refinement and Development subsection.

## Results

### Study Participants

#### Characteristics of Study Participants

In total, 8 participants completed the study: 5 (63%) men and 3 (38%) women. Their average age was 56 (SD 13.79; range 28-74) years. Of the 8 participants, 6 (75%) were Chinese, 1 (13%) was Malay, and 1 (13%) was White. Participants varied in their highest level of education, with 50% (4/8) having a postsecondary-level education, 38% (3/8) holding a university degree, and 13% (1/8) having a primary- or secondary-level education. Regarding employment status, 6 (75%) were full-time employees, 1 (13%) was self-employed, and 1 (13%) was unemployed. Regarding marital status, 5 (63%) were married, 2 (25%) were single, and 1 (13%) was widowed. Regarding annual household income, 3 (38%) had between S $3000 (US $2232) and S $5999 (US $4464), 3 (38%) had >S $6000 (US $4465), and 2 (25%) had <S $3000 (US $2232). Of the 8 participants, 6 (75%) were living with a spouse or family member, 1 (13%) with a helper, and 1 (13%) was living alone.

#### Study Participant Health Profile

The average age at diabetes diagnosis was 43.6 (range 24-59) years. Half (4/8, 50%) of the participants were taking oral medication, insulin, or traditional medicine for diabetes, whereas the other half (4/8, 50%) were not following any treatment plan for diabetes. Of the 8 participants, 5 (63%) reported managing the disease independently, whereas 3 (38%) reported that their spouse or a helper supported them in managing the disease. Three-fourths (6/8, 75%) of the participants experienced no diabetes complications, whereas 25% (2/8) reported single or multiple complications such as retinopathy, diabetes-related ulcerations, neuropathy, sexual problems, and hypoglycemia. In terms of the frequency of physician consultations, 5 (63%) reported visiting their physician 2 to 3 times a year, 2 (25%) reported 1 visit a year, and 1 (13%) reported 1 visit every 2 years.

#### Diabetes Knowledge

Regarding their diabetes knowledge, 4 (50%) rated it as *above average*, 3 (38%) as *moderate*, and 1 (13%) as *excellent*. Of the 8 participants, 3 (38%) had attended diabetes self-management education, whereas 5 (63%) had not participated in diabetes self-management education and were not recommended to do so by their physicians. When asked where they usually sought diabetes knowledge, 4 (50%) reported using search engines, 3 (38%) used social media (eg, YouTube or Facebook), and 1 (13%) attended courses or seminars.

#### Health Attitudes and Beliefs

All participants stated that they were open to receiving health advice from others, and 88% (7/8) disagreed with the statement “My diabetes self-care is poor.” Of the 8 participants, 7 (88%) strongly agreed that regular check-ups are necessary for maintaining good health and moderately or strongly agreed that adherence to prescription drug regimens is beneficial. Additionally, 5 (63%) stated that they would see a physician immediately if they felt ill or noticed something very unusual or disturbing happening with their bodies.

#### Use of Technology

All participants reported that they had no trouble using mobile phones and were either somewhat or extremely comfortable with mobile apps. Participants used apps daily for a variety of reasons: 6 (75%) used apps for social networking; 4 (50%) for entertainment as well as food and drink; 3 (38%) for finance, health and fitness, and news; 2 (25%) for lifestyle and sports, photo and video sharing and documentation, and shopping; and 1 (13%) for other purposes, such as education, books, business, productivity, and utilities. The majority of the participants (5/8, 63%) stated that they would be willing to use an app if it was prescribed by a health care professional. Almost all (7/8, 88%) believed that a mobile app could help them manage diabetes, and 50% (4/8) had already used at least 1 app for this purpose, primarily for blood glucose or medication tracking. Of the 4 participants who had already tried a mobile app to manage their condition, 3 (75%) agreed that their behavior had changed as a result of using the app and that they had observed improvements in eating habits and adherence to the prescribed medication plan. The features that participants believed were lacking in previously used apps were the ability to communicate with other devices and provision of feedback in insulin dosage calculators.

### Thematic Analysis

On the basis of the interview responses, we devised deductive and inductive themes that are presented in Textbox 1.

**Research objectives and themes identified based on analysis of the interview transcripts.**

**Challenges of living with type 2 diabetes**

- Solitary accountability
  Taking ownership
  Anchoring motivation
- Translating knowledge into practice
  Localization and personalization
  Trial and error
- Pragmatic self-awareness
  Area of control
  Overcoming oneself

**Validation, acceptability, and usability of the prototype**

- Information is the key
- The power of appearance
- Ambivalence and polarization toward chatbot
- Potential impact
  Me versus them mindset
  Potential power users

**Perspectives on digital diabetes education**

- Information-seeking strategies
  Self-sufficiency
  Critical analysis
- Desired content
  General approach
  Topics
  Formats
- Desired qualities
  Accessibility
  Adaptability
  Autonomy
  Evidence-based design and content
  Gamification
  Guidance
  Integration
  Personalization
  Up-to-date content

#### Challenges of Living With T2D

##### Solitary Accountability

*Taking ownership* of the condition is the first step toward transformation and better diabetes management. Building a sense of accountability and taking the condition seriously is a solitary act that requires a transition from playing a passive role to playing an active role, where one strategizes and adjusts one’s priorities, does one’s own research, and puts a conscious effort into bringing about change instead of counting on others to solve one’s problem:

Okay. I think the most important thing I would maybe say is...ownership. Yes, I have met many people fellow diabetics and it boils down to ownership or the lack of it, ok?...you’re either the player or the victim, you know, right? When you want to play the victim: “Oh, I have diabetes and talked to the doctors, the doctors said I can do this, I can’t do that.” But if you want to be a player, you take charge. The doctor is just your support, the doctor is a support system; now, you take charge of your own condition, you make sure you have things, strategies put in place, things that you have to do, you know? Ways to change your diet, your lifestyle and stuff like that, right? And that’s where a lot of, many people fail because...“So, I’m a diabetic, you know?” [impersonating an attitude of victimhood]. I just wait for my 3-month doctor’s appointment, and then the doctor says, “Okay, you’re not doing very well let’s increase your meds.” So, it’s like you’re just being the victim. And it’s not helping, and you just sliding down the slope, and eventually just reach rock bottom.

I1, participant 7

*Anchoring motivation* together with taking ownership of the condition is crucial to initiate the shift toward healthy behavior. A person with diabetes may be driven either by an unwelcome vision of the future or the opposite: aspiring to a positive future goal. One may wish to save oneself from the likelihood of a reduced quality of life, exemplified by an increased number of clinic appointments, growing medical bills, limited mobility, or general health problems. Others may prefer to focus on something to look forward to, such as seeing their children grow up or enjoying their hard-earned money. Furthermore, emotions such as fear, frustration, and desperation or, alternatively, a sense of duty to serve as role models for peers who live with the same condition can also serve as a motivator:

I think, it’s more on my health rather than other thing. Because [indistinguishable] if you have a lot of money, but your health is no good, it’s useless at all. Basically, it’s my health. The main thing that triggered me to keep on [indistinguishable] is the health.

I1, participant 9

##### Translating Knowledge Into Practice

*Localization and Personalization* means adapting information to make it relatable and actionable. The diabetes journey requires individuals to put effort into translating health advice to specific circumstances on the one hand and translating their experiences to others on the other hand. Besides the difficulty of personalizing specialized medical advice from physician appointments, health education leaflets, or medical books, study participants pointed out how challenging it is to adapt Western-centric advice to the local context. Available resources often lack an Asian perspective, which can be problematic for residents of Singapore, whose cuisine, for example, is a mix of Malay, Indian, and Chinese influences. This can result in confusion caused by incompatible or conflicting advice:

To use the local word in Singapore, is rojak [which means eclectic mix in colloquial Malay], it means, it’s all...it’s all in my mind: plant based, local food, you can eat rice, balanced meal...so, many, many ways, I don’t know, some days I just swing this side, some days I just swing this side; it’s not working, I’ll just say okay let’s go back to base one.

I1, participant 10

Moreover, individuals living with diabetes may need to translate their experiences to others, particularly when traveling to countries where awareness of the condition is low. In other cases, they may need to convince health providers that their body responds nontypically to standard treatments and that they require a personalized approach. They may also need to build self-confidence and learn how to express themselves to overcome close community skepticism when, for example, starting a new diet.

Once individuals take ownership of their condition, identify the source of motivation, and translate medical advice into an actionable and personalized plan, they begin the process of modifying their daily routines through *trial and error*. This involves accepting the possibility of mistakes, setbacks, and failures and requires perseverance, discipline, and attentiveness. Patients may encounter astonishing learning moments while experimenting with their own bodies, such as how the body reacts to certain food or how the sense of taste changes and helps them transition to new diets. The trial-and-error approach also comes with unexpected challenges; for example, a long walk in hot tropical weather may cause the skin of the feet to peel intensely. Experimenting with one’s own body requires proactiveness but can be supported by surrounding circumstances, as in the case of a study participant with >40 years’ experience of living with T2D who only recently experimented with different types of carbohydrates:

Yeah. I’ve cut rice almost entirely. I used tapioca and all those other things, taste good but they don’t like me very much. Right? So, I found out a lot during the lockdown just by being able to experiment. How much I could eat and... not make the rise blood sugar wild. I’ve changed my diet quite a bit during the COVID period. Said, no more rice, very limited amount of anything else that’s made of carbohydrates, and of course no sugars or anything like that.

I1, participant 2

##### Pragmatic Self-Awareness

*Area of control* signifies the extent to which a person has the power to take decisions that impact one’s life. Understanding what is within one’s locus of control and acknowledging one’s weaknesses is crucial in cultivating self-compassion and avoiding disappointment; for instance, recognizing one’s lack of cooking skills may prompt one to become more cautious while eating out. External barriers beyond one’s control may also require solutions; for example, if a continuous glucose monitoring system is too expensive, one can perform time-constraint experiments. Similarly, if one is planning to travel to areas with restricted access to insulin, one can prepare additional insulin doses. Realizing one’s locus of control supports building resilience and regulating emotions, contributing to self-compassion:

Do what you can within your own means, yeah, and therefore, you will become very realistic, you become more pragmatic. Being pragmatic, and therefore you also manage expectations; when you set yourself expectations that you cannot meet you will only result in being, you know, it will only result in you being disappointed

I1, participant 7

*Overcoming oneself* is a process of understanding one’s limitations in order to find a new way forward. Once the area of control has been identified, individuals can begin working toward overcoming negative attitudes and harmful behaviors. This process requires addressing skepticism or objections toward new solutions and changes, such as overcoming reluctance toward wearing orthopedic shoes, which are considered unaesthetic, or taking insulin, which is considered complicated. Overcoming oneself may involve doing something that one dislikes, such as reducing carbohydrate intake by cutting down on favorite dishes, drinking tea or coffee without sugar, avoiding condiments, or eating out less frequently. When it comes to physical activity, this could include adding exercise to one’s daily routine:

[Indistinguishable name] she has this walking exercise, so this time around I’m so determined; so, I put on: going to salad, measuring my sugar, monitoring my calories, I’m also putting in half an hour of exercise. So, you see. A lot of work.

I1, participant 10

A unique cognitive obstacle that the participants faced was overcoming unconscious incompetence, which requires recognizing skill or knowledge deficits; for example, the realization that one uses binge eating to cope with uncomfortable emotions may force one to decide to take control of the condition. Alternatively, one can acknowledge a lack of cooking skills and decide to learn to cook to follow a specific diet.

#### Validation, Acceptability, and Usability of the Prototype

Overall, the acceptance of the app was positive. Three-fourths (6/8, 75%) of the participants declared that they would use the app in the future and recommend it to others.

##### Information Is the Key

**Figure 2:**
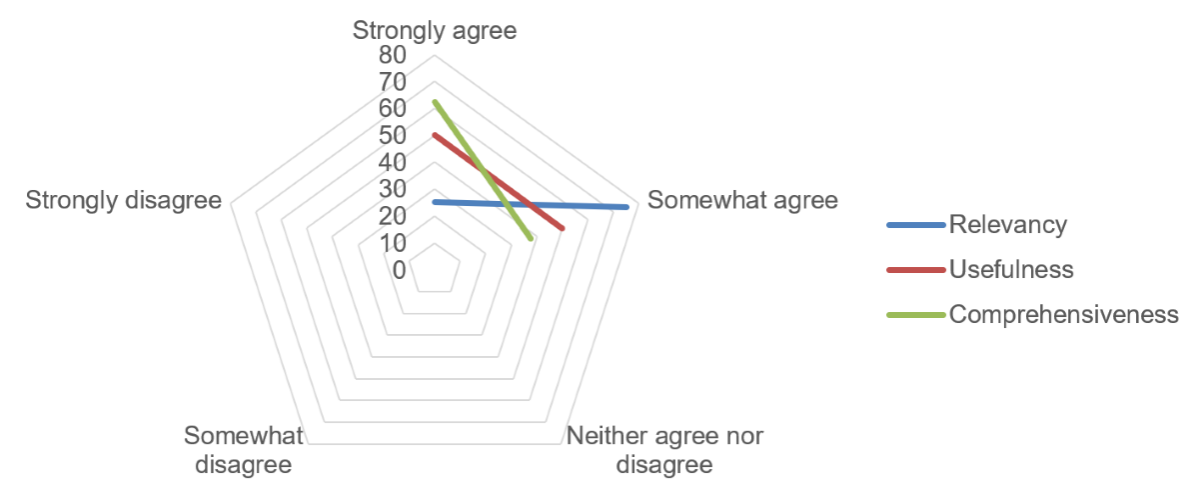
Perceived relevancy, usefulness, and comprehensiveness of the app content in percentages.

The educational content was well received, with high acceptability. The delivery method suited 38% (3/8) of the participants strongly and 50% (4/8) moderately, whereas 1 (13%) of the 8 participants remained neutral. During the interviews, participants highlighted that the proportion of text and images was appropriate and that the visuals prompted them to read the text. They described the educational content as “useful,” “informative,” “bite sized,” “relevant,” “interesting,” “easy to understand,” “comprehensive,” “important,” and “complementary to what the doctor mentioned.” Survey data showed that the content was perceived as relevant, useful, and comprehensive (Figure 2):

It’s just informative, easy to understand. It’s not so technical that, know, you...you stop reading or...yes, it’s quite informative, is very easy to understand from diabetic point of view.

I1, participant 4

When asked if they would save the educational content on their mobile phones for future reference, of the 8 participants, 3 (37%) indicated that they would surely do so, and 3 (37%) would consider it, whereas 2 (25%) remained neutral. Regarding acceptance of the quizzes, 25% (2/8) of the participants liked them a great deal, and 50% (4/8) somewhat liked them, whereas 25% (2/8) remained neutral. However, during the interviews, 1 (13%) of the 8 participants expressed disapproval of the quizzes, saying that they felt unnecessary. The acceptance of microchallenges was identical to that of the quizzes: 25% (2/8) of the participants liked them a great deal, and 50% (4/8) liked them somewhat, whereas 25% (2/8) remained neutral. Of the 8 participants, 1 (13%) suggested an additional option of skipping the microchallenges, especially because there are no rewards for completing them.

When asked if they liked the self-reflection card, half (4/8, 50%) of the participants agreed, whereas the other half (4/8, 50%) remained undecided. Similarly, in terms of attitudes toward review cards, designed in a way that is analogous to web-based product reviews, half (4/8, 50%) of the participants remained neutral. During the interviews, participants suggested that the notification content should be functional, including a prompt to read or continue reading a module with an estimated time to finish reading it, rather than a personal or knowledge-based message:

Is it a good time...oh, maybe you can include a time given for the module...is this a good time to complete this 3-minute module? And they will be more interested...I only need 3 minutes, maybe I can do it over my train or waiting for my food, something like that. I think that would be helpful.

I2, participant 1

Notifications could also be used for spaced learning. Most of the participants (6/8, 75%) preferred to receive notifications no more than once a week, whereas 13% (1/8) preferred once a day, and 13% (1/8) said never. The willingness to be contacted by methods other than push notifications (ie, email or telephone call) when the app had not been accessed for a while was mixed, with 50% (4/8) of the participants agreeing and 38% (3/8) disagreeing.

##### The Power of Appearance

Half (4/8, 50%) of the participants strongly agreed that the dashboard was concise and displayed their progress clearly; they also strongly agreed that the brightness and contrast of the dashboard were appropriate. Of the 8 participants, 3 (38%) strongly accepted the color palette, whereas 3 (38%) moderately accepted it, and 2 (25%) remained neutral. Half (4/8, 50%) of the participants strongly believed that the arrangement and size of buttons, icons, menus, and content on the screen were appropriate, whereas the other half (4/8, 50%) expressed moderate approval. With regard to usability and accessibility for motor or visual impairments, 63% (5/8) of the participants declared that there was somewhat sufficient space in the interface to minimize touch errors, whereas 25% (2/8) strongly agreed, and 13% (1/8) remained neutral.

On the basis of the survey data, all participants were satisfied with the app’s appearance. Half (4/8, 50%) of them expressed a strong positive stance, especially in the context of learning cards, where they appreciated that “it’s not gimmicky.” Interestingly, when the same question was asked in the context of the chatbot prototype, the proportion of participants with a strong positive stance reduced from 50% (4/8) to 38% (3/8). The perception of the chatbot prototype’s appearance seemed to be associated with the perception of the feature’s functionalities. The participant who most strongly opposed the use of a chatbot evaluated its appearance as “kitsch.” Furthermore, when asked about the app’s branding, exemplified by the product name and a hero mascot depicted in an icon, most of the participants were neutral (5/8, 63%), whereas 25% (2/8) liked it, and 13% (1/8) disliked it. However, additional comments in the survey suggested that the participants had difficulty understanding the story behind the branding. Similarly, during the interviews, the relatability of the icon in the branding was raised:

I find it quite relevant, and it’s quite interesting, except for the one that look like Superman, I don’t like that.

I2, participant 4

##### Ambivalence and Polarization Toward the Chatbot

During the interviews, participants shared ambivalent and polarizing opinions on diabetes education conveyed in the format of a chatbot conversation. Some of the participants (6/8, 75%) were more accepting, stating that it was more engaging and interactive than the educational module, felt friendly, and provided more personalized care, which would motivate them to act. On the other end of the spectrum, some of the participants (2/8, 25%) viewed the chatbot unfavorably owing to the predictability and rigidness of a chatbot conversation without free-text input, exacerbated by previous negative experiences with chatbots used by institutions such as banks. In these cases, the user felt like a passive conversation participant and awaited in anticipation of being contacted by a human:

Although if you try to make it look like it’s more interactive, it’s not...because it’s merely asking me for inputs. For it to proceed to the next tab I have to put in an input, yes or no, black or white, picture number 1 or picture number 2. So, I would consider myself as a passive participant in that.

I2, participant 7

In the surveys, 50% (4/8) of the participants strongly agreed that they would use the chatbot in the future, whereas 25% (2/8) somewhat agreed, 13% (1/8) somewhat disagreed, and 13% (1/8) remained neutral. One-fourth (2/8, 25%) of the participants stated that they would definitely recommend the chatbot to others, whereas one-fourth (2/8, 25%) were slightly less willing, and half (4/8, 50%) were moderately willing to do it. When asked about their willingness to pay for the services of the app based on the prototype with textbook-like diabetes education, half (4/8, 50%) of the participants expressed mere possibility (“maybe”), whereas the other half (4/8, 50%) were unwilling. However, when participants were asked the same question in an altered context—seeing a broader view of the functionalities, including chatbot conversations beyond frequently asked questions—the degree of unwillingness to pay for the services decreased from 50% (4/8) to 25% (2/8). However, there were some doubts raised about the ways in which the app would maintain long-term engagement:

How can my interest and usage in the Apps continue? If it is all educational I can get bored after sometime.

S2, participant 4

##### Potential Impact

Using survey data, we confirmed participants’ perceptions of the app’s potential impact. Of the participants, 63% (5/8) somewhat agreed that the app could increase their likelihood of a better quality of life, 25% (2/8) strongly agreed, and 13% (1/8) remained neutral. When asked whether using the chatbot would make it easier to manage their diabetes, the majority of the participants (6/8, 75%) agreed, whereas 25% (2/8) remained neutral. Regarding enhancing the app by adding a chatbot function, 63% (5/8) of the participants strongly agreed that it could increase the chances of having a better quality of life, 25% (2/8) somewhat disagreed, and 13% (1/8) moderately agreed. Interview data revealed two subthemes that addressed the potential impact of the low-fidelity prototype of the app: (1) *me versus them* *mindset* and (2) *potential power users*.

**Figure 3:**
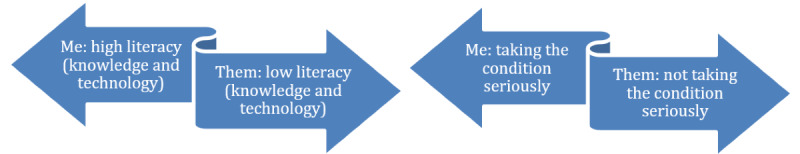
Subthemes for the me versus them mindset.

The *me versus them mindset* subtheme revolves around contrasting oneself with other people with diabetes and can be further split into 2 focal points: literacy and attitude (Figure 3). The literacy-based *me versus them* *mindset* was exemplified by the participants through statements comparing themselves with other people with diabetes and asserting that the latter do not have sufficient diabetes knowledge to manage the condition well or sufficient technological literacy to acquire proper know-how. In addition, according to the study participants, other people with diabetes do not present a proper attitude toward the condition because they do not take it seriously, refuse to get educated, or prefer a passive approach and medication dependency. Nevertheless, the participants highlighted that they would gladly recommend the app to others who, unlike themselves, have lower diabetes literacy and a lower willingness to learn and manage the condition:

Well, myself, I’d probably introduce it to other people who don’t know it. I have a lot of experience; I still have both of my feet and all of my toes. That is not accidental. Right? But I know people who are di... diagnoses as diabetic but don’t take it seriously until things go wrong.

I1, participant 2

*Power users* are usually defined as those who show the most advanced computer skills or those who interact with the product the most compared with other customers. In this study, we use this term to describe potential leading users of the product, the ones that are the most willing to get familiar with the app and are excited about it. The study revealed 3 potential power users who exhibited high excitement about, and interest in, the app to address their personal needs. The potential power users exhibited enthusiasm about the relatability of the content that was higher than that of others, declared their intention to repeatedly go through the same content, or performatively interacted with the app—when asked in the chatbot conversation to check their toenails, they stopped reading to bend down slightly and look at their feet:

It is actually referring to me.

I1, participant 4

If I were to go through this module, definitely I’ll go through 3-4 times.

I1, participant 4

#### Perspectives on Diabetes Education

##### Information-Seeking Strategies

*Self-sufficiency* was one of the skills identified by the participants as crucial in obtaining relevant diabetes-related information. This is essential owing to the solitary nature of the diabetes journey and the constant need to understand and manage the condition. People living with diabetes must be self-sufficient and proactively search for information on their own even in the absence of immediate health issues. This process of inquiry is ongoing and facilitated by the internet (eg, Google or YouTube), medical books, or experiments.

*Critical analysis* is the ability to evaluate the quality of evidence and determine when it can and can’t support one’s position. All of the study participants exhibited an active approach to seeking diabetes information, such as finding research studies to participate in, attending diabetes outreach programs, or consulting with specialists. This behavior might not be representative of the whole Singaporean community of people with diabetes. It’s important to note that the participants were recruited over the web through diabetes-focused social media groups, which may imply internet savviness. In addition, participants highlighted the importance of developing the skill of filtering out irrelevant or erroneous information. This includes coming up with strategies for effective search queries; identifying reliable sources, including discerning credible sources from advertisements or misinformation; and determining what information corresponds to personal circumstances:

There are a lot of things that are aimed at a particular kind of problem don’t really apply to you. Right? So, I find most of the things online are related to non–insulin-dependent diabetes. Right? And things that you might be able to control or would work for a little while, I’ve tried that too. Again, it had an effect for about 2 weeks, it’s totally useless after that. Right? There’s a lot of stuff that you have to learn to filter out.

I1, participant 2

##### Desired Content

Participants shared the desired features and qualities that they would look for in an ideal diabetes education app. Textbox 2 summarizes the list of desired content in an app, in addition to that already present in the prototype.

**Desired content in a diabetes education app on top of that already present in the prototype.**

**General approach**

- Localized examples
- Habit-oriented and “how-to” strategies
- Advanced knowledge

**Topics**

- Diet
- Drug interactions
- Physical activity
- Others: dental health, traveling, interactions with other conditions, and thick skin on feet

**Formats**

- Instructional videos
- Pictures in the conversation
- Realistic photos

Participants preferred localized examples; habit-oriented strategies or *how-to*–style advice; and the inclusion of more advanced, less obvious, and less common information (eg, the Somogyi phenomenon, ie, a hypoglycemia episode overnight that leads to hyperglycemia in the morning owing to a surge of hormones [54]).

*Topics* of the highest interest primarily consisted of information on diet (including the consumption of local food such as durians as well as strategies for managing weight, controlling food intake, and overcoming binge eating) and strategies to become physically active. Among other topics, participants desired to know about drug interactions; ways to deal with challenges during travel (eg, preparing more insulin or tips to overcome uncertainty about the ingredients in foreign cuisine); debunking myths; learning more about dental health; interactions with other health conditions or aspects of health such as mood and appetite; and dealing with issues that are not yet serious, such as thick skin on feet.

Additional desired informational formats included instructional videos, pictures in the chat conversations, and realistic photos of complications to help in self-assessment.

##### Desired Qualities

Textbox 3 summarizes the list of desired qualities in an app, aside from those already present in the prototype.

Participants highlighted the need for increased *accessibility* (eg, voice-overs as well as adjustable font size and contrast, and image size). For users with visual or motor impairments, simple user interfaces are necessary to reduce errors. In addition, to reduce the digital divide, the app’s features should be simplified for less technology-savvy users, modified to accommodate the lower attention span of older adults, and made multilingual for a broader application in Singapore’s multiethnic population.

Participants underlined the need for *adaptability and personalization*. Adaptability involves adjusting specific content, feedback, and advice based on the user’s habits, needs, and level of literacy. Personalization entails adjusting the app’s features and content to the user’s characteristics, including the severity of their condition, age, sex, and use preferences:

I think you can have option. Either 1 or 3 [conversation prompts in each unit] or whatever you see. ’Cause, uh, different educational level, different age, and things like this, so they might, you know, find you know...For those who are not so well educated they’ll find it a bit tough. And those are of reasonable educated, then you know, too easy for them and things like that. So they defeat the purpose.

I2, participant 5

When the interviews focused on chatbot conversations, *autonomy* was one of the key qualities mentioned. Participants craved the option to go back to rerun a conversation; the freedom to search topics; and, most often mentioned, 2-way interaction with a free-text input option because, as they stated, they do not like to be told what to do:

But... I mean, when you said chatbot, okay, I deal with a lot of chat bots so, for example, you know, like nowadays, most of the telcos, the mobile service providers, they are running on chat bots. You hardly actually connect to a real person unless you have really some heavy duty problem which the chatbot gets overwhelmed. But this chatbot is very... I mean, apart from requiring me to give inputs, right? I mean, it’s just [indistinguishable] it’s just option. So, I do not have the option to type something; so, maybe, your chatbot, I mean, AI is... I mean, you guys didn’t factor in, like, you know, that people ask questions.

I2, participant 7

**Desired qualities in a diabetes education app on top of those already present in the prototype.**

**Accessibility**

- Adjustable font size, image size, and contrast
- Simplified user interface to reduce errors
- Simplified features
- Voice-overs, including in the chatbot
- Multilingual functionalities

**Adaptability**

- Adapting to user’s habits
- Adapting to user’s needs
- Adapting to user’s level of literacy

**Autonomy**

- Going back in a conversation
- Search option in the conversation
- Free-text conversation

**Evidence-based content**

- Indication of the authors and references
- Guarantee of information validity

**Gamification**

- Incentives
- Time frame to complete a module

**Guidance**

- Personal diary
- Medication management tool
- Carbohydrates calculator
- Exercise tracking
- Blood glucose level calculator

**Integration**

- Embedded in the process of care
- Chatbot connected to a human

**Personalization**

- Condition severity
- Age
- Sex
- Notification timing or use preferences

**Up-to-date content**

- New medical knowledge
- Daily insights

Participants also highlighted the importance of *guidance*, which can be achieved through personalized feedback and recommendations embedded in a personal diary or in medical management tools. In addition, guidance can be designed within carbohydrates calculators or exercise and blood glucose level (BGL) calculators to warn users about how the intensity of planned exercise will affect the BGL or whether the planned meal exceeds the allowed carbohydrate intake limit:

And then, let’s say, if I key in some exercises: “It could help me reduce blood sugar at this level.” Sort of that. That would be very useful. That is something that I’m looking for.

I1, participant 4

The app and content are expected to be evidence based. In addition, evidence of the app’s effectiveness should be provided. This emphasized the unmet need of patients for a reliable and trustworthy tool to provide them with information about their condition. The app should also be regularly updated with the most recent medical knowledge and disseminate the information through notifications to encourage user engagement.

Some of the participants (3/8, 38%) expected *gamification* features, including incentives (even if they were only symbolic) or a time frame to complete a module, to boost app engagement.

Finally, participants suggested 2 approaches for *integrating* the app into diabetes management and the pathway of care. One approach is to incorporate the app into the patient care process by involving physicians in the app recommendation process to provide additional motivation to patients to use the app. Alternatively, the app can be integrated with other technologies, such as BGL monitoring systems, and provide educational content tailored to individual results.

### App Prototype Refinement and Development

The conclusions drawn from the analysis of surveys and interviews have influenced the development of the app. Subsequently, the app will be tested in a feasibility study and randomized controlled trial. Table 1 summarizes the solutions that have been implemented to address the desired qualities of the app and better serve end users. However, owing to the scope of the app developer’s work as described in the vendor contract and budget constraints, some of the design suggestions put forward by the participants could not be implemented in the next version of the app. Table 2 summarizes the ideas that have been put on hold.

**Table 1:** Solutions implemented to address desired qualities during app development.

Domain	Details	Solutions implemented
Accessibility	Adjustable font size, image size, and contrast Simplified user interface to reduce errors Simplified features Voice-overs	Choice of font size and theme (dark or light) in the onboarding and profile Zooming in on the images in the text after clicking on them A simple dashboard with 3 main buttons and a bottom button ribbon (Recommended for You, Continue Learning, Already Completed; Profile, Library, Search) Audio added to each learning card (generated using Text To Speech Free web-based software [55])
Adaptability	Adapting to the user’s needs Adapting to the user’s level of literacy	The user is asked “What single change do you think would improve your life right now?” The question concerns a change that would have an impact on the first recommended module (diet, exercise, medication, or introduction) After clicking on a module, the user is asked 2 knowledge-based questions and 1 behavior-based question related to the topic of the education module. If the user answers all 3 questions correctly, they are informed that they can skip reading the module
Autonomy	Going back in a conversation Search option in the conversation	A back button was added to the conversations Search option added in conversations and for education modules
Evidence-based design and content	Indication of the authors or references Guarantee of information validity	Each module introduction includes information about the authors (including our clinical partner) Each summary includes lists of references based on which the content has been devised
Gamification	Time frame to complete a module	Each unit has a description that includes the estimated time needed to complete it
Guidance	Personal diary	Selected units include a question on actionable conclusions that the user came up with after reading the content
Integration	Embedded in the process of care	A platform for physicians where they can check the activity of the user (completed modules) has been developed
Personalization	Notification timing or use preferences	Notification preferences added to the onboarding and settings
Up-to-date content	New medical knowledge Daily insights	Administrator-controlled notifications with new content developed

**Table 2:** List of desired qualities not implemented in the app development.

Domain	Details	The rationale behind not implementing other desired qualities
Accessibility	Voice-overs in the chatbot Multilingual functionalities	Developing functionality in the chatbot builder for automated voice-over generation (budgetary constraints) Developing a multilingual app would require additional efforts (budgetary constraints)
Adaptability	Adapting to the user’s habits	The solution would require developing a complex framework for passive and active data collected in the app (timeline constraints)
Autonomy	Free-text conversation	The solution would require resource-intensive training in AI^a^-based tools such as Rasa for free-text conversations. Free-text automated conversations may be prone to faulty health advice, and the provenance of the advice may be unclear (AI “black box”)
Gamification	Incentives	The solution would require either additional financial capability or intense efforts to build partnerships that would provide vouchers
Guidance	Medication management tool Carbohydrates calculator Exercise tracking BGL^b^ calculator	The solutions would require developing separate passive and active data collection modules in the app (budgetary constraints); they would also require developing complex evidence-based rules for feedback, taking into consideration individual health constraints
Integration	Chatbot connected to a human	The solutions would require hiring trained diabetes consultants available daily for the users (budgetary constraints)
Personalization	Condition severity Age Sex	The solutions would require developing additional app modules that would show different content according to a selected variable (budgetary constraints)

^a^AI: artificial intelligence.

^b^BGL: blood glucose level.

## Discussion

### Principal Findings

This study used a co-design approach that involved 8 potential end users in testing to improve the design and content of a low-fidelity diabetes education app prototype. The aim of the study was to gain insight into the challenges of living with T2D, examine perspectives on the use of the app, and analyze attitudes toward digitalized diabetes education. Participants emphasized the importance of realizing the individual nature of accountability required in health decision-making to enable healthy living with diabetes. They stressed the need for accountability, taking ownership of one’s health condition, and discovering personal motivators for change, which are vital to being proactive in one’s solitary health journey. This process requires assuming responsibility for translating health advice to one’s circumstances and accepting that the process is iterative. Once individuals realistically understand the areas within their control and recognize the need to go beyond their comfort zone, a lifestyle change can occur.

The potential impact of the diabetes education app prototype was expressed by identifying the subthemes of potential power users and the *me versus them* *mindset*, with the former referring to users with the highest engagement in the app and the latter referring to contrasting oneself with other people with diabetes who do not realize the importance of the condition and do not demonstrate sufficient knowledge.

Regarding perspectives on diabetes education, participants recognized the necessity of acquiring critical analysis skills, including self-sufficiency to search for relevant diabetes information and the ability to discern between reliable information and false information. Desired app content included localized examples; habit-focused strategies; advanced knowledge; and formats such as realistic photos, instructional videos, and pictures in the conversations. Desired qualities included accessibility, adaptability, autonomy, evidence-based design and content, gamification, guidance, integration, personalization, and up-to-date content. The co-design study demonstrated that involving end users to evaluate a low-fidelity app prototype and content and contribute to improving it can result in efficient and concrete discussions about possible solutions. The findings allowed us to improve the design of the app as well as its features and content before releasing it to a wider audience in a trial.

### Comparison With Prior Work

Previous research recommends considering patients’ preferences, beliefs, and values when validating the product designed for them, suggesting co-design as one of the methods for developing interventions that prioritize inclusion and shared decision-making [56]. Similar to other co-design studies, we were able to improve our understanding of the characteristics of people living with T2D and of the desired characteristics of technology solutions that could empower them [57]. Our findings correspond with prior studies exploring the facilitators of behavior change that list self-discipline, described by our participants as accountability and ownership; the fear of complications; and education as the pillars of lifestyle changes among Singaporeans [58]. Creating an impact on attitudes, values, beliefs, and emotions should not be ignored in health education interventions [59] because high health literacy may not translate into a healthy lifestyle [60]. Moreover, high diabetes literacy among people living with diabetes in Singapore [60] could explain the *me versus them* *mindset* discovered among the participants of this study, which emphasizes highly favorable knowledge self-evaluation in comparison with peers. Adding on to other studies that highlight the desire to learn more, especially about diet and physical activity [61,62], our co-design emphasizes the need for localized content; solution- and habit-focused approaches; and advanced, less obvious information to maintain user interest.

In terms of key app qualities, Dhinagaran et al [63] recommend personalization, suitable frequency and duration, ease of use, engagement, and relevancy to the target population. Our proposed list includes accessibility, adaptability, autonomy, evidence-based design and content, gamification, guidance, integration, personalization, and up-to-date content. These concepts, especially adaptability, complement personalization and information design constructs in the digital patient experience framework, including profiling, tailoring, and autonomy [64]. In a co-design study for a T2D self-management app, Kwan et al [65] found that people with diabetes in an Asian context expect reminders and notifications for medication, integration with glucometers and blood pressure machines, a local food database, 1-touch logging of medication and food, and an export function for sharing data with physicians. Our findings confirm that the self-management component, including tracking features, is a desired feature. However, it should be enhanced by providing informative and personalized feedback on the data entered, including the estimated consequences of planned exercise or meals. The diabetes education app prototype tested in this study, rather than focusing on self-management support through data collection and tracking, aimed at broadening the knowledge and skills of people living with T2D through educational material, quizzes, and conversations. Incorporating tracking features has been planned for the future phases of the app’s development because providing additional value to the pathway of care by digitalizing diabetes education and making it interactive has been considered fundamental. However, it has to be acknowledged that tracking features, as opposed to educational features, carry a higher potential of maintaining user attention and engagement.

Regarding the integration of the app into the regular pathway of care, our study participants expressed a preference for incorporating the app into the clinical pathway rather than providing in-app social support from peers. This is in contrast to the study on multiethnic Singaporeans by Yoon et al [62], which found that in-app social support was preferred, and the study by Tay et al [66], which found that social connectivity can promote collective lifestyle behavior change. In addition, Jeffrey et al [67] identified a lack of health care professional recommendations on the use of self-management apps as one of the barriers to their use, which might be related in part to the health care professionals’ concerns regarding their own preparedness and workload capacity [68]. Our findings suggested receptivity toward digital therapeutics prescribed by physicians, which aligned with the advocacy in the study by Anderson and Emmerton [69] for combining app interventions with medical check-ups, rather than relying solely on mobile devices. Our findings might be an early sign of receptivity toward digital therapeutics prescribed by physicians [68]. In addition, several studies on mobile health services have shown that performance expectancy is one of the determinants of the intention to use digital health tools. This highlights the need for further evaluative studies, including randomized controlled trials, to confirm the app’s effectiveness, which we plan to conduct in the postdesign phase.

### Strengths and Limitations

The co-design approach of this study involved end users in the design process, whose feedback is invaluable in compiling recommendations on the desired content and qualities of digital diabetes education tools. However, recruitment proved to be a challenge, and, as a result, the generalizability of the findings is limited by the small sample size. Another limitation arises from the nature of web-based recruitment, which resulted in gathering insights from participants with a high level of technology literacy, which might not be representative of the Singaporean community of people with diabetes. Another challenge encountered during the study was the unfamiliarity of participants with the concept of a flat prototype with limited interaction options and preset flow, leading to some difficulty in validating the prototype. Although the concept was explained to participants, their expectations of full functionality resulted in a partially abstract experience. Despite these limitations, the study adds to existing research by highlighting the importance of localized content; habit-focused and *how-to* approaches; and advanced, less obvious information to maintain user interest. Furthermore, the proposed app qualities of accessibility, adaptability, autonomy, evidence-based design and content, gamification, guidance, integration, personalization, and up-to-date content should be seen as a strength of the study in terms of assisting future development of diabetes education apps.

### Conclusions

The last decade has seen an increase in the development of health apps that support diabetes self-management, and this trend is expected to continue. The aim of this study was to investigate the acceptability and usability of a diabetes education app prototype among people living with T2D and its potential impact on their diabetes self-management skills and practice and quality of life. The study found that the app was acceptable to most of the participants (6/8, 75%) and suggestions were made for possible improvements. The changes made in the subsequent iterations of the prototype during app development included, among others, adding an adaptive learning component that allows tailoring the education pathway to the user’s knowledge and needs. This study provides evidence for the design and development of an accepted tool for diabetes education and can inform future digital diabetes education interventions.

## Appendix

Multimedia Appendix 1 Screenshots of the app prototype.

Multimedia Appendix 2 COREQ (Consolidated Criteria for Reporting Qualitative Research) 32-item checklist.

Multimedia Appendix 3 Interview questions.

Multimedia Appendix 4 Codebook for qualitative analysis.

## Abbreviations

A1: aim 1
A2: aim 2
A3: aim 3
BGL: blood glucose level
COREQ: Consolidated Criteria for Reporting Qualitative Research
I1: interview 1
I2: interview 2
HITAM: health information technology acceptance model
MARS: Mobile Application Rating Scale
S1: survey 1
S2: survey 2
S3: survey 3
S4: survey 4
T2D: type 2 diabetes

## Data Availability

Deidentified interview data are available on request. Researchers who wish to obtain further details may contact the corresponding author with a methodologically sound proposal.
