# Supplementary material for: A Diabetes Education App for People Living With Type 2 Diabetes: Co-Design Study
Source: JMIR Form Res. 2023 Sep 18;7:e45490. doi: 10.2196/45490 (PMC10546275; doi:10.2196/45490)
Supplement: Multimedia Appendix 1 [file formative_v7i1e45490_app1.pdf]

Appendix 1: Screenshots of the app

Mock-ups of the app prototype

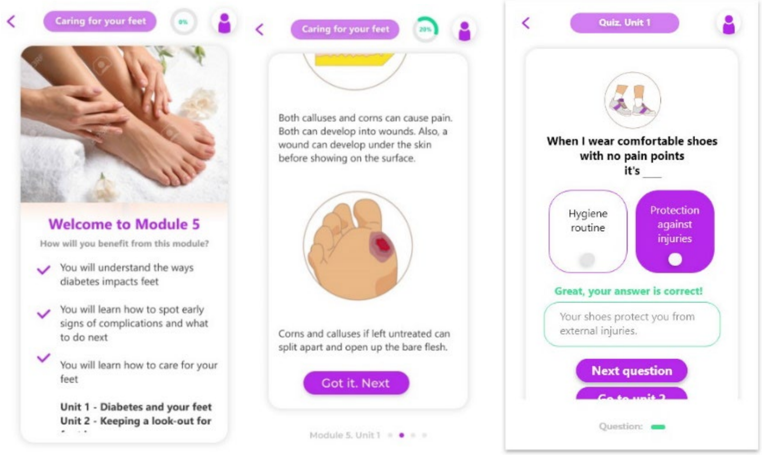

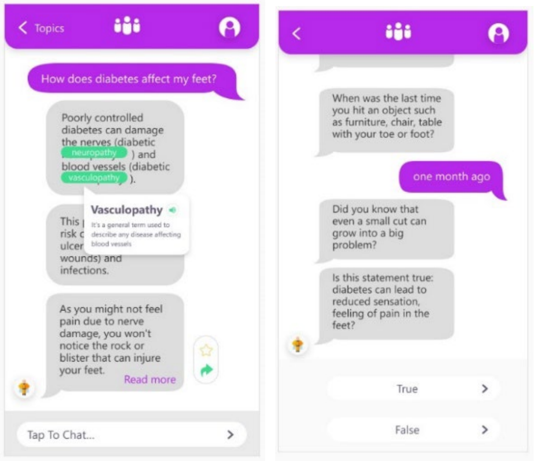

Conversations in Adobe XD prototype

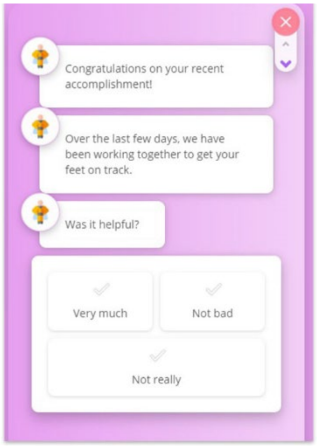

Quriobot conversation prototype
