# Supplementary material for: A Diabetes Education App for People Living With Type 2 Diabetes: Co-Design Study
Source: JMIR Form Res. 2023 Sep 18;7:e45490. doi: 10.2196/45490 (PMC10546275; doi:10.2196/45490)
Supplement: Multimedia Appendix 3 [file formative_v7i1e45490_app3.pdf]

Appendix 3: Interview questions

Interview 1

Did you like or dislike the app? What did you like? What did you dislike? Why?

Can you think of a moment when someone gave you useful advice on diabetes self-management, you have put the advice into practice, and it turned out to be both helpful and effective?

Think of a situation when you have managed to solve a problem related to diabetes on your own. What was it? Why do you think it happened? What did you do? What did you think? How did you come up with the solution? Was anyone else involved?

Interview 2

Interview guide/questions

What were your thoughts and feelings while testing? Was it engaging?

How do you like the conversations? What do you dislike about them? How could it be better?

Do you feel that they would impact your daily life, your behavior?

How do you feel about receiving notifications? Would you like to be able to set the time or rather be surprised by a notification?

Would you be open to receiving one notification per day, per week, per month? How often would you agree to receive a notification that an education module is waiting to be completed? What about health tips or system notifications?
