# Supplementary material for: A Diabetes Education App for People Living With Type 2 Diabetes: Co-Design Study
Source: JMIR Form Res. 2023 Sep 18;7:e45490. doi: 10.2196/45490 (PMC10546275; doi:10.2196/45490)
Supplement: Multimedia Appendix 4 [file formative_v7i1e45490_app4.pdf]

Appendix 4: Codebook for qualitative analysis

Deductive theme	Inductive theme	Description	Code	Quote	Reference
		Strategies that enable a proactive approach	Anchoring motivation	You'll find out "ay! this is helpful", you want to try it for yourself, and you have any those results that show it on your body is very helpful and then yeah you'll be very happy that you have reversed the condition.	I1 subj 1
	1.1. Solitary accountability		Taking ownership	Okay. I think the most important thing I would maybe say is… ownership. Yes, I have met many people fellow diabetics and it boils down to ownership or the lack of it, ok? So, I have diabetes… [mocking someone] you know, you're either the player or the victim, you know, right? When you want to play the victim: 'Oh, I have diabetes and talked to the doctors, the doctors said I can do this I can't do that.' But if you want to be a player, you take charge. The doctor is just your support, the doctor is a support system, now, you take charge of your own condition, you make sure you have things, strategies put in place, things that you have to do, you know? Ways to change your diet, your lifestyle and stuff like that, right? And that's where a lot of many people fail because… 'so, I'm a diabetic, you know?' [impersonating an attitude] I just wait for my three-month doctor's appointment' and then the doctor says 'okay you're not doing very well as increase your meds'. So, it's like you're just being the victim. And it's not helping and you just sliding down the slope, and eventually just reach rock bottom.	I1 subj 7
1. Challenges of living with diabetes	1.2 Pragmatic self-awareness	Strategies to increase chances of lifestyle change	Area of Control	My mathematical skills were challenged. Yeah those are probably the major highlights or lowlights, I guess. For the first 15 to 20 years, I gradually started cutting things out of my diet; I	I1 subj 2

				found alcohol particularly give very unpredictable results. So, I think, by the late 80s, I had stopped drinking entirely.	
			Overcoming oneself	Well, when previously, I used to drink, maybe three, four cups of tea a day, now I cut down to one or two cup and it's teh [‘tea’] without sugar. No more ordinary tea. It's more evaporated milk tea without sugar. And I go for whole meal bread more as much as I can.	I1 subj 4
	1.3. Translating knowledge into practice	Strategies that a person with diabetes must implement to make medical advice useful for them	Localization and personalization	So there's these two sides pulling each other, oh I am so [a gesture of being overwhelmed] oh and I'm Asian, so my diet... and these people are Westerners, so, the food and my Asian food, it's very conflicting, very conflicting, and so I will say that my struggle is ongoing.	I1 subj 10
			Trial and error	I found that the COVID lockdown or whatever you want to call it, last year, was quite good because it gave me some time to experiment with what I was eating. I found, for instance, that potatoes have virtually no effect on my blood sugar.	I1 subj 2
2. Validation, acceptability and usability of the diabetes education app prototype	2.1 Information is the key	The biggest value is the usefulness and comprehensiveness of information		So, you have bite-sized and then after that they have like a bit of… like a quiz along the way, right? So that somewhat keeps the interests. That is very important to me. Because when, I mean, nobody wants or likes to be diagnosed as a diabetic [laughs] and then you make them go through an app and then they have to read like tons of things… it will just turn them off, so making it bite-sized, interesting, you have quiz along the way… it helps to somewhat nudge them to want to know more, I mean, that's how I feel it is.	I1 subj 7

	2.2 The power of appearance	The perception of appearance is connected with the perception of usefulness		I find it quite relevant and it’s quite interesting, except for the one that look like Superman, I don’t like that.	I2 subj 4
	2.3 Ambivalence and polarization toward chatbot	Perceptions of the usefulness of the chatbot are either ambivalent or polarized.		Although if you try to make it look like it's more interactive, it's not… because it's merely asking me for inputs. For it to proceed to the next tab I have to put in an input, yes or no, black or white, picture number one or picture number two. So, I would consider myself as a passive participant in that. So, it's not so helpful per se, lah. The purpose of the chatbot should be an extension, an option for the person, the users to clarify and to find out more. Yeah, it's, I mean maybe as an end-user who…	I2 subj 7
	3.3. Potential Impact	Evaluation of the degree the app would be helpful for the participant	Me vs Them	I still have both of my feet and all of my toes. That is not accidental. Right? But I know people who are diet diagnoses as diabetic but don't take it seriously until things go wrong.	I1 subj 2
			Potential power user	Okay, what I like about this module is… it is actually referring to me, okay? Caring of feet? Yes, I don’t care. Because sometimes we don't go into it, you are lazy.	I1 subj 4
3. Perspectives on diabetes education	3.1. Information-seeking strategies	Approaches to acquiring relevant information	Critical analysis	For me, like for example, for me when I got the diabetes, I would go to the internet to search all over theme, look for information about diabetes. Yeah but I think most internet things… sometimes the information may not be correct.	I1 subj 9
			Self-sufficiency	Mostly from what I learn from the lectures and also from Internet. Yeah so like I search YouTube they have those like, diabetic association which tells you which are the best food to eat and best food to avoid. Then from there, I just pick and choose what to put in my diet, and suit my daily lifestyle.	I1 subj 10

	3.2. Desired content	App content that would make the tool more useful	General	Yes, so which shoe, shoe a or shoe b, shoe a is more diabetic friendly, shoe b is diabetic-friendly, but shoe b is a $100, shoe a non-diabetic is $25. Now, that's a real decision, you know?	I1 subj 7
			Topics	So, food-related questions are something that are always useful, particularly when I'm also Warfarin. Is it okay to eat durian? I love durians. I found: one piece I can get away with, two pieces not advisable.	I1 subj 2
			Format	all of that could be, you know, an instructional video on how to care for your nails. It would be one way.	I1 subj1
	3.3. Desired qualities	App qualities that would make the tool more useful	Accessibility	You know, I have my fat fingers, and it would take a long time for me to type, so, I just call you, you know yeah, so, in that…	I2 subj7
			Adaptability	For me personally I think I will not be slight… I think it's still ok. It's still okay, especially for some people who don't have any knowledge about this and want to know further. Yes, probably for those who already got some ideas about these, I don't think, it's necessary for them,	I2 subj9
			Autonomy	For people who are not very patient maybe they wouldn't find it very helpful. Maybe there are people who just want to scroll a lengthy… an article and then just to skim through it.	I2 subj1
			Evidence-based	Yeah so with all this information that this app is providing, how well does it help in managing my glucose level in my body? Otherwise, if you give me all this information, if I do it all	I1subj8
			Gamification	If there is a reward in completing… some form of the reward, then it will be more… and it would be more incentivized to complete the module. If not, for the person itself it's just a meaningless module, and if they do not have the interest to learn.	I2 subj1

			Guidance	So, how much I take, it’s after I’ve taken it, it will be useful if, let’s say, immediately after I take so much of this large, maybe fried rice and: “what ey, you have taken too much, be careful of this, be careful of that”. I mean sort of some indication that…. I’d be interested in this sort of app.	I2 subj4
			Integration	so the question is who will access this. Is it compulsory. Or it's only meant for those people who would search for it, or it auto pop in like, for example, like, for example, I’m pre diabetic right, so I have, I can show you we have so many different types of apps, will I consciously go to look for this app? Unlikely, unless I face a problem. And so. Unless this education is tied in somehow is tied into pre visit or post visit; that means, for example, today I’m going to see the doctor to follow up for my routine and then there's the SMS messages that "hey you know, can you just click on link today"; or something along that line as a prompter. Because you don't prompt me, I will, I will not even think about this platform is available for me to go look for it. And to me, would be um Do I need it?	I1 subj 10
			Personalization	Uhm the quiz, okay, so, so, you've noticed that I, I hold for a moment and I thought to understand the question diabetes-friendly.... So it takes a while to process that; it's very simple, one was a flat shoe, one was a high heel, but I'm a female, I can relate, but what if it is a male	I1 subj10
			Up-to-date	By the way, do you have any intention to input like something like new medical knowledge, technologies on diabetes, maybe there's some medicine or some new finding on the diabetes.	I2 subj 9
